# Extracellular Matrix-Based Biomaterials for Cardiovascular Tissue Engineering

**DOI:** 10.3390/jcdd8110137

**Published:** 2021-10-22

**Authors:** Astha Khanna, Maedeh Zamani, Ngan F. Huang

**Affiliations:** 1 Graver Technologies, Newark, NJ 07105, USA; akhanna@gravertech.com; 2 Department of Cardiothoracic Surgery, Stanford University, Stanford, CA 94305, USA; maedeh@stanford.edu; 3 Stanford Cardiovascular Institute, Stanford University, Stanford, CA 94305, USA; 4 Department of Chemical Engineering, Stanford University, Stanford, CA 94305, USA; 5 Veterans Affairs Palo Alto Health Care System, Palo Alto, CA 94304, USA

**Keywords:** tissue engineering, regenerative medicine, extracellular matrix (ECM)

## Abstract

Regenerative medicine and tissue engineering strategies have made remarkable progress in remodeling, replacing, and regenerating damaged cardiovascular tissues. The design of three-dimensional (3D) scaffolds with appropriate biochemical and mechanical characteristics is critical for engineering tissue-engineered replacements. The extracellular matrix (ECM) is a dynamic scaffolding structure characterized by tissue-specific biochemical, biophysical, and mechanical properties that modulates cellular behavior and activates highly regulated signaling pathways. In light of technological advancements, biomaterial-based scaffolds have been developed that better mimic physiological ECM properties, provide signaling cues that modulate cellular behavior, and form functional tissues and organs. In this review, we summarize the in vitro, pre-clinical, and clinical research models that have been employed in the design of ECM-based biomaterials for cardiovascular regenerative medicine. We highlight the research advancements in the incorporation of ECM components into biomaterial-based scaffolds, the engineering of increasingly complex structures using biofabrication and spatial patterning techniques, the regulation of ECMs on vascular differentiation and function, and the translation of ECM-based scaffolds for vascular graft applications. Finally, we discuss the challenges, future perspectives, and directions in the design of next-generation ECM-based biomaterials for cardiovascular tissue engineering and clinical translation.

## 1. Introduction

Vascular diseases such as atherosclerosis, aortic aneurysm, and peripheral arterial disease are a major cause of mortality and loss of quality of life [1]. The replacement of damaged or diseased vasculature with engineered substitutes is a rapidly advancing pursuit of researchers in the field of vascular tissue engineering and regenerative medicine. One of the challenges is to engineer three-dimensional (3D) vascular tissues that have structural integrity and that support physiological function [2]. Extracellular matrix (ECM) is secreted by cells and is composed of structural and regulatory proteins and polysaccharides. Each organ and tissue is composed of a distinct ECM with respect to its biochemical composition and structural organization. In particular, the vascular ECM consists of biomolecules such as collagen, laminin, fibronectin, elastin, and heparan sulfate proteoglycans [3]. The ECM regulates many cellular functions such as proliferation, migration, and differentiation [3]. Since ECM-based biomaterials can mimic the composition and structure of the ECM, they are attractive biomaterials for repairing or restoring damaged organs and tissues. The ECM plays a vital role in vascular tissue engineering by providing both a physical scaffold for structural integrity and 3D shape, as well as relaying intrinsic biochemical and mechanical cues that regulate cellular function.

There has been tremendous progress in the field of vascular tissue engineering and regenerative medicine in the past decades, notably with the development of biomaterials derived from ECM proteins that provide mechanical support and biochemical signals that modulate vascular cell attachment, phenotype, and behavior. ECM-derived biomaterials were initially used as two-dimensional (2D) coatings for the improved cell adhesion to tissue culture polystyrene dishes. Later advancements led to three-dimensional (3D) ECM-derived biomaterials with improved tunability to recapitulate the dynamics, composition, and structure of the native ECM [4].

Vascular cells are responsive to the ECM in a process known as dynamic reciprocity [5] or bi-directional cross talk between the cells and their environment [6,7,8], which is accompanied by the continuous and dynamic remodeling of the ECM [9] into tissue-specific 3D architectures and compositions. In turn, the ECM sends mechanical and biochemical cues to the resident vascular lineages through the engagement of cell-surface receptors, the activation of intracellular signaling cascades, and the intrinsic changes in gene expression and cell phenotype [2,6]. The synthesis and secretion of ECM molecules by vascular lineages in physiological and pathological [10] conditions regulates numerous biological processes, including vascular differentiation [11,12], angiogenesis [10,13], and wound healing [14,15,16]. By virtue of its evolutionary conserved composition and its impact on embryonic development, cellular, and organ homeostasis, the ECM constitutes an ideal substrate to promote the repair of damaged or diseased tissue, and also as serves as a scaffold for engineering whole tissues and organs. The past decade has had remarkable progress towards the next generation of ECM-based biomaterials, including advancements in the isolation and characterization of ECM components, as well as the development of decellularization techniques to preserve native mammalian structure and composition.

In this review, we highlight major developments in biomaterials derived from mammalian ECM in the field of vascular tissue engineering and regenerative medicine, with a focus on translational applications. We also discuss the efficacy of ECM-derived biomaterials to regenerate and repair tissues, along with the progression of therapies from in vitro to preclinical studies, and ultimately towards clinical translation.

## 2. Overview of the Vasculature

The human circulatory system consists of a sophisticated network of blood and lymphatic vessels that transport fluids throughout the body [17,18]. Each level of hierarchy plays an important role in maintaining homeostasis throughout the body. Large vessels such as arteries and veins conduct the transport of large volumes of blood between organ systems. Large vessels progress into smaller vessels to control blood pressure and volumetric flow to the cells and tissues in an organ. Large and small vessels work together to maintain homeostasis throughout the body. Capillaries are the smallest and most densely distributed vessels, and they have a specialized role of directly exchanging fluid with cells in the tissues, as well as being involved in lymphocyte migration and homing [17,18,19]. The distribution and orientation of the microvasculature is influenced by the metabolic activity of the specific tissue [20].

**Figure 1:**
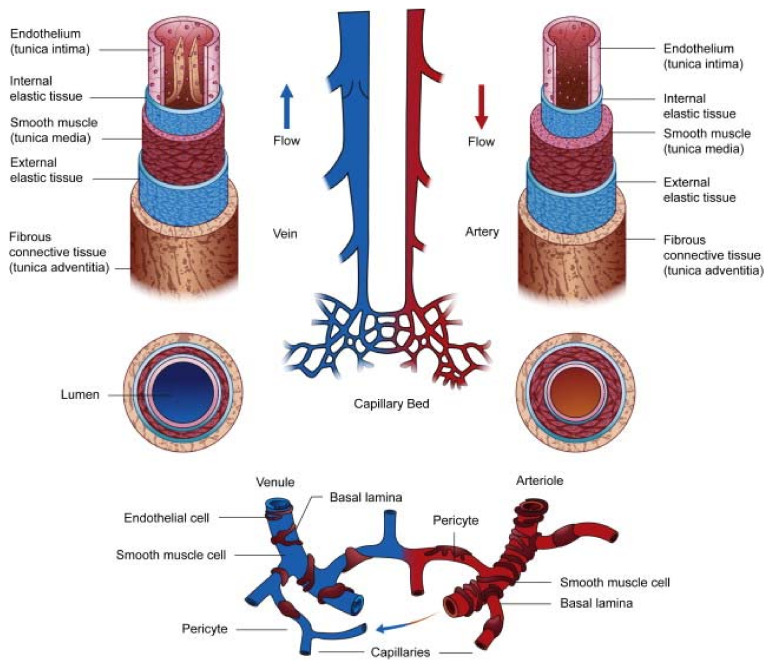
Anatomy of large and small blood vessels. Reprinted from Stratman et al. [21]. “Chapter 24-Blood Vessel Formation” from *Principles of Developmental Genetics (Second Edition),* with permission from Elsevier.

The anatomy of large and small vessel differs in structure and function (Figure 1) [20,21]. Large vessels (i.e., arteries and veins) are composed of three layers: an inner layer composed of endothelium; middle layer formed by smooth muscle cells, elastic tissue, and collagen fibers; and outer layer composed of elastic tissue and collagen fibers [22]. The percentage of elastic tissue in the arteries in considerably higher than veins because arteries transport blood at higher pressures [23]. Small vessels (i.e., arterioles, venules, and capillaries) are narrower and thinner than large vessels and participate in blood perfusion. Capillaries are the smallest vessels and are one-cell thick to allow mass exchange and fluid permeability. The cellular structure of large and small vessels is also distinctive. Arteries, veins and arterioles are composed of endothelial cells (ECs), smooth muscle cells (SMCs), and pericytes [24]. Venules are made of ECs, pericytes, and SMCs that differ in vessel thickness that than arterioles [25,26]. Capillaries are composed of a single layer of ECs and pericytes [19].

ECs form the innermost layer of the vasculature. These cells are highly polarized in which their luminal side is exposed to blood circulation, while their basal side is anchored to basal membrane produced by ECs themselves. ECs are responsible for vasomotion (i.e., contraction–relaxation of vessel wall) by secretion of vasoactive cytokines and response to circulatory vasomotion mediators. ECs also play a vital role in thromboregulation, inflammatory responses, and vascular permeability [27]. Vascular SMCs are predominantly responsible for maintaining the integrity of the vessel and providing mechanical support for contraction. These cells are actively involved in the secretion of ECM components, including collagens, elastins, and proteoglycans [28]. Similarly, pericytes are perivascular cells that wrap around vessels and play an SMC-like role in stabilizing the vasculature, while also contributing to angiogenesis [29].

Despite the difference in the structural and functional aspect of various blood vessels, the ECMs found in blood vessels generally consist of a similar ECM composition. The ECMs in the basement membrane upon which the ECs reside include laminin, collagen IV, nidogen, and heparan sulfate proteoglycan [30]. The combination of the biochemical and physical cues, provided by the self-assembled layer of proteins that form the basement membrane, are major elements of the vascular EC microenvironment [30]. The basement membrane compliance and topography are intrinsic attributes of the EC microenvironment [30]. Besides the ECMs found in the basement membrane, the ECMs found in other parts of blood vessels include elastin that provides elasticity and collagen for structural integrity.

## 3. ECM-Based Biomaterials

The vascular system performs a critical role in maintaining homeostasis by carrying out the transport of oxygen, nutrients, blood cells, and hormones through continuous blood circulation in the body. Blood is transported to all parts of the body through blood vessels. Owing to these specialized functions of the vasculature, vascular biomaterials should exhibit characteristics of tissue resilience and structural mimicry, while supporting cellular function. There has been tremendous progress in the production of ECM-based biomaterials in the past two decades. Numerous studies [31,32] have utilized the components of native ECMs as a hydrogel for efficient encapsulation, while providing tissue-like water content.

To modulate mechanical properties of ECM-based hydrogels, various physical and chemical crosslinking methods have been employed. Physical crosslinking approaches such as ionic interaction (crosslinking between two molecules with opposite charges), hydrogen bonding, thermal induction-mediated solid-to-gel phase transition, and chemical approaches (i.e., photo-polymerization, enzymatic crosslinking) have been employed [33]. Crosslinking conditions such as light exposure sites, intensities, and periods can modulate the mechanical properties of ECM-based hydrogels [34,35].

Among physical crosslinking approaches, hydrogels are ionically crosslinked under mild temperature and physiological pH conditions [36]. Hydrophobic interactions result in polymer swelling by water uptake that forms the hydrogel. Alginate, a polysaccharide derived from seaweed, can be crosslinked at room temperature and physiological pH, making it a frequently used ECM for the encapsulation of living cells [37]. Polysaccharides such as chitosan and dextran have been used to produce hydrogels by hydrophobic interactions [36]. In another example, freeze/thaw temperature change induce the formation of poly (vinyl alcohol) (PVA) hydrogel through the formation of PVA crystallites that act as crosslinking sites in the network [37]. Additionally, poly(acrylic acid) and poly (methacrylic acid) form a complex with polyethylene glycol (PEG) by hydrogen bonding as a result of the pH dependent swelling of the gels [38]. Capello and collaborators employed protein interactions to crosslink silk-like and elastin-like block co-polymers using hydrogen-bonded beta sheets or strands. A major advantage of physical crosslinking methods is the absence of harsh reagents that supports cellular encapsulation within the hydrogel, but a disadvantage is the limited range in the hydrogel’s mechanical strength.

Aside from physical crosslinking methods, chemical crosslinking such as chain growth polymerization (free radical, cationic or anionic) involves the processes of initiation, propagation and termination. Radical polymerization of low molecular weight monomers in the presence of crosslinking agents can be used to product chemically crosslinked gels. For example, poly(2-hydroxyethylmethacrylate) (pHEMA) hydrogels are formed by polymerization of HEMA in the presence of crosslinking agents such as ethylene glycol dimethacrylate [39]. For example, Sperinde et al. employed transglutaminase to synthesize PEG-based hydrogels [40]. Although chemical crosslinking is versatile and can be used to product hydrogels with good mechanical stability, the crosslinking agents can be toxic compounds and can react with the bioactive compounds to product adverse reactions. This is avoided in the physically crosslinked gels. The incorporation of other ECM components can also be used to enhance the mechanical integrity of ECM hydrogels such as network formation between individual ECM components (i.e., collagen/fibrin [41,42], fibrin/HA [43], collagen/chondroitin sulfate/ hyaluronic acid (HA)) [44]. The incorporation of ECM components has not only been shown to enhance mechanical integrity, but it also modulates cellular behavior such as in the upregulation of tissue specific gene expression and ECM molecule secretion [43,44,45].

Commonly, ECMs are isolated from human or animal tissues [46]. A popular method for biopharmaceutical application is recombinant protein secretion using Chinese hamster ovary cells [47]. These artificial or ECM-like proteins are of great interest in biomaterial scaffolds as they can have a specific sequence and a defined molecular weight [48,49]. Additionally, owing to the popularity of basement membrane ECM derived from Engelbreth–Holm–Swarm (EHS) mouse sarcoma cells, some ECMs can also be isolated from this basement membrane matrix. Finally, ECMs can also be derived by decellularization of tissues for subsequent recellularization. The properties and in vitro applications of common ECM-based biomaterials such as collagen, elastin, and decellularized ECM are described below.

**Collagen:** Collagen is the most abundant structural protein in the mammalian ECM that imparts tensile strength to prevent deformation of tissue [50]. Collagen provides mechanical integrity to the vasculature and other biological tissues. Structurally, collagen is composed of three parallel polypeptide strands that form a triple helix, and the arrangement of varying polypeptide strands lead to a large number of isoforms. Among them, collagen I and collagen III isoforms are found in the tunica media and tunica adventitia, respectively. Collagen-based biomaterials can be developed using several strategies. Among them, collagen can be purified from common tissue sources such as calf skin or rat tail to form a functional scaffold. Alternatively, collagen-based scaffolds derived from decellularized matrix preserve the original tissue shape and ECM structure.

Collagen-based biomaterials from extracted collagen have been employed to study cell behaviors such as vascular EC migration, proliferation, differentiation, and phenotypic expression [51,52,53,54,55]. We previously demonstrated using arrayed ECM microenvironments that human induced pluripotent stem cell (iPSC)-derived ECs cultured on multi-component ECMs containing purified collagen IV had a positive impact on cell viability in hypoxia, as well as in supporting nitric oxide production and phenotypic marker expression of CD31 [56]. Scaffolds derived from purified collagen I enhanced the survival of ECs derived from induced pluripotent stem cells for treatment of peripheral arterial disease in a murine model [57]. In addition, some studies suggest that collagen I is involved in the biological and electrophysiological function of the myocardium [58,59,60], and therefore it has been used extensively in cardiovascular regeneration applications [58,61]. Schenke-Layland et al. showed that collagen type IV coatings promote induced pluripotent stem cell differentiation into cardiovascular and hematopoietic lineages [62]. These examples highlight the utility of purified collagen as a biomaterial for modulating cell function and survival. To better reproduce the characteristic of cardiovascular tissue ECM, several researchers have used collagen in combination with a fibrous protein, fibrin. Fibrin mitigates the risk of immunological incompatibility, and its biodegradable along with tunable physical properties makes it a suitable ECM protein for tissue engineering and regenerative medicine. Fibrin possesses both elastic and viscous properties, and incorporation with fibrin has been shown to enhance physical property and increased tissue compaction, a morphogenetic process improving structural tightness [63].

**Elastin:** Another essential ECM component that provides elasticity to the cardiovascular tissue is elastin, which is involved in cellular functions such as cellular attachment, proliferation, differentiation, and migration [64]. Wang et al. demonstrated that collagen–elastin hydrogels support the proliferation and protein expression of valve interstitial cells and valve ECs providing enhanced physical strength and elasticity [65]. Edalat et al. developed a hybrid hydrogel consisting of components Matrigel^TM^, type 1 collagen, and growth factors that showed enhanced cardiomyocyte differentiation.

**Decellularized extracellular matrix (dECM)**: Decellularization is the process of removing cells and cellular debris from a tissue or organ and isolating the extracellular matrix (ECM). The decellularized ECM contains proteins, proteoglycans, and glycosaminoglycans responsible for cell adhesion, cell remodeling, and mechanotransduction. Importantly, dECMs do not contain many immunogenic components that are found in the native tissue, making them attractive for cardiovascular tissue engineering and regenerative medicine applications. In addition, tissue-specific dECMs also retain the biochemical and structural properties necessary for tissue function [66,67]. The general guidelines for acceptable amounts of residual DNA after decellularization are <50 ng dsDNA per mg dry weight and <200 base pair DNA fragment length. An optimized decellularization process efficiently removes the cellular components of ECM but also preserves the microstructural, biomechanical, and biochemical properties of ECM. A combination of different mechanical, chemical, and enzymatic decellularization methods is commonly used to obtain suitable dECM for intended application.

Several studies have conducted optimization of decellularization methods using physical, chemical, and biological treatments [68,69,70]. McFetridge et al. developed a two-step decellularization process of porcine carotid artery using various solvents and trypsin to extract the lipid and cellular proteins, respectively. They and demonstrated that the composition of the solvents used for lipid extraction significantly affected the dECM modulus, concluding that an ethanol–butanol–ethanol three-step extraction preserves the mechanical properties of dECM, comparable to that of control tissue [71]. Apart from decellularization process, the tissue origin, age, and species were also found to be important in specific applications, due to the role of these factors in the microstructure, mechanical properties, and composition of ECM. Sellaro et al. showed that sinusoidal ECs maintained their phenotype in culture longer when seeded on liver dECM compared to the ECM derived from bladder or small intestine [72]. Although dECMs derived from the corresponding tissues of non-human sources might not be ideal for clinical applications, they still hold promise to be translated into the clinic, particularly for cardiovascular regeneration. This is due to their similar microstructure, mechanical properties and composition, and greater abundance, compared to that of human dECM. The overall microstructure of ECM remains unchanged by aging. However, the composition and structural organization of ECM components, particularly proteins, undergo substantial changes over time, which further modulates the mechanics of ECM as well as cellular responses [73].

dECM exhibits great potential for the regeneration of engineered cardiac and vascular tissues. dECM has been widely used owing to the preservation of natural bioactive molecules that foster homeostasis and facilitate the tissue regeneration process [69,74,75,76,77]. One of the seminal papers in dECM translation is the work by Ott et al., who developed a bioartificial tissue engineered myocardium with a preserved ECM composition using coronary perfusion-based whole organ decellularization of rat hearts, followed by recellularization with neonatal cardiac cells and rat aortic ECs [77]. The constructs exhibited contractile function. Cardiac tissue-derived ECM has also been shown to promote in vitro differentiation and maturation of cardiomyocytes derived from human embryonic stem cells or human induced pluripotent stem cells [78,79]. Bosara et al. developed hydrogels using decellularized human myocardium-derived ECM with gelatin methacryloyl (GelMA) or GelMA-methacrylated hyaluronic acid (MeHA). The hydrogels supported the growth of human induced pluripotent stem cells derived cardiomyocytes (iCMs) and human cardiac fibroblasts (hCFs) [79]. Similar approaches have also been taken to decellularize blood vessels [80], and the decellularized vessels have been shown to be safe for in vivo implantation [81].

Mesenchymal stem cells (MSCs) have been widely reported to promote in vitro and in vivo angiogenesis [82]. Bone marrow-derived MSCs have shown success in clinical applications for angiogenesis in cardiovascular diseases like critical limb ischemia [83]. It has been shown that both adult bone marrow-derived and adipose-derived MSCs can stimulate angiogenesis [84]. MSCs derived from prenatal and adult tissues have also shown the potential for immunomodulatory function [85] and hematopoietic support [86]. MSC-derived ECMs have also been shown to have angiogenic potential [87,88,89]. MSCs and ECs in co-culture deposited decellularized ECM deposited by the co-culture of MSCs and ECs stimulated an angiogenic response by facilitating cross talk through paracrine and juxtacrine cellular interactions between MSCs and ECs [89]. Yael et al. fabricated hybrid ECM-based hydrogels using decellularized porcine cardiac extracellular matrix (pcECM) and demonstrated that these hydrogels are naturally remodeled by MSCs, supporting cellular viability, morphology, and organization. The hydrogels exhibited no in vitro or in vivo immunogenicity. Further, in a rat model of chronic myocardial infarction (MI), the pcECM-based hydrogels enabled improvement in cardiac function 12 weeks post MI [90]. The effects of ECM-based biomaterials are summarized in Table 1.

The biological response of cardiovascular tissue to dECM scaffolds is summarized in Table 2.

**Cell Culture-Derived ECM:** Decellularized ECMs derived from tissues have been used as biomaterial scaffolds in tissue engineering. They have the advantage of maintaining the structure of their respective tissues and organs. However, concerns of limited autologous tissue/organ availability, immune responses, and the risk of pathogens from allogenic and xenogeneic tissues have led to the rise of cell culture-derived ECM scaffolds [106]. Cell culture-derived ECM can be developed into 3D scaffolds or reconstituted with natural or artificial polymers for tissue engineering applications [107,108,109]. Cultured cells have advantages over tissues such as the following. Cultured cells can be maintained in pathogen free conditions. Cell-derived scaffolds can provide the accurate geometry and porosity and optimal cell penetration that is difficult to achieve in tissue derived scaffold. In vitro cultured cell types can be used to combine their respective ECMs. Furthermore, autologous cells can generate their own ECM that obviates immune responses. Lu and colleagues developed ECM scaffolds derived from MSCs, chondrocytes, and fibroblasts by culturing cells in a poly(lactic-co-glycolic acid) (PLGA) template [110]. The ECM scaffolds promoted cell proliferation, cell adhesion and ECM production. Fibroblasts cultured in the fibroblast-derived ECM scaffolds proliferated and produced ECM to fill the pores of the scaffold. In 2 weeks of culture, a multi-layered tissue was produced with homogenously confluent fibroblasts [110].

## 4. ECM Regulation of Vascular Function and Cell Fate

Blood vessels carry out a critical role in blood circulation and have layers of structures of varied structure and function. They have a complex structure of with layers of thick and elastic walls (tunica intima, tunica media, tunica adventitia). The tunica media consist of SMCs that provide contractility, whereas the tunica adventitia provides connective tissue support [111]. The tunica intima, an essential part of blood vessel, consists of a monolayer of ECs that constitute the interior surface and basal lamina that encloses it. The basal lamina contains type IV, XV, and XVIII collagens; laminin; fibronectin; heparan sulfate proteoglycan; and other macromolecules [112]. ECs attach to the basal lamina and degrade it when initiating angiogenesis. Laminin is the most abundant protein in the basal lamina and serves as the foundation for the protein network and supports migration, proliferation, and differentiation of ECs [113]. Nguyen et al. demonstrated that laminin supports differentiation of human embryonic stem cells into functional endothelial progenitor cells [114]. Stamati et al. showed that laminin facilitates the 3D in vitro vascular network formation in collagen hydrogels by promoting the uptake of vascular endothelial growth factor by ECs [115].

The endothelial basement membrane is comprised of a milieu of different ECMs such as laminin, type IV collagen, fibronectin and heparan sulfate proteoglycans [116]. It is well recognized that the endothelial basement membrane provides signaling cues for physiological endothelial behavior and function [117]. These biochemical and physical cues modulate endothelial cell adhesion, migration, proliferation, differentiation, and the cellular signaling network. The biochemical and physical cues also participate in the angiogenesis process [117]. During angiogenesis, the basement membrane is degraded by matrix metalloproteinases (MMPs) limiting the presence of type IV collagen. The ECM, with a high laminin content, stretches out resulting in its increased compliance that promotes migration and proliferation of endothelial cells [117].

Multi-component ECM scaffolds have shown potential to promote iPSC-EC viability, endothelial phenotype, and nitric oxide production under hypoxia [118]. Using an arrayed ECM microenvironment platform, we previously showed that multi-component ECMs generally improved endothelial differentiation of human induced pluripotent stem cells, compared to single-component ECMs. Combinatorial ECMs such as collagen IV + gelatin + heparan sulfate + laminin and collagen IV + fibronectin + gelatin + heparan sulfate + laminin were shown to significantly improve cell survival, nitric oxide production, and CD31 phenotypic expression, compared to single-component ECMs. Among them, the collagen IV-containing multi-component ECMs supported the endothelial differentiation, based on multifactorial bioinformatic analysis [118]. This approach reveals ECM interactions and cellular behavior in response to complex environmental cues that cannot be exhibited by conventional cell culture platforms especially under conditions such as hypoxia and nutrient and growth factor deficiency.

Biodegradable ECM-based biomaterials related to the basal lamina including collagen and fibronectin have been utilized for studying EC function. Integrin-dependent binding to RGD cell adhesive regions on collagen nanofibrils or fibronectin reinforces endothelial sprouting [119,120]. Daum et al. showed that fibronectin coating on synthetic vascular grafts supports endothelium formation [121]. RGD-modified HA hydrogels seeded with ECs improved cell proliferation, migration, and capillary-like network formation, while promoting the formation of functional vasculature after in vivo implantation [122]. In another example, endothelial progenitor cells encapsulated in blood vessel derived dECM exhibited higher proliferation capability and enhanced vascular network formation, compared to collagen hydrogel [123]. These studies illustrate the ability of ECM-based biomaterials to modulate endothelial behavior, including their organization, proliferation, and sprouting. Table 3 illustrates different ECM-based biomaterials and their impact on endothelial growth, differentiation, and function.

## 5. Considerations of Hydrogel Assembly, Biofabrication, and Spatial Patterning

**3D Hydrogel Assembly:** ECM hydrogels have been one of the major advancements in the engineering of ECM-based biomaterials. Hydrogels are injectable and compatible with several fabrication technologies including 3D printing, micropatterning, and electrospinning, thereby expanding the clinical applications of ECM-based biomaterials. Hydrogels are hydrated polymers or materials with ≥30% (*v*/*w*) water content that use crosslinks between their constituents to maintain their structural integrity [131]. Some ECMs can self-assemble to form hydrogels such as collagen, elastin, hyaluronic acid (HA), and alginate [132]. Other hydrogels are commonly made of synthetic [133] polymers such as poly (vinyl alcohol) (PVA), poly (acrylic acid) (PAA), polyethylene oxide (PEO), and poly(propylene fumarate-co-ethylene glycol) [P(PF-co-EG)]. Synthetic hydrogels can also be formed from proteins with biomimetic cell adhesive ligands such as RGD (from fibronectin) and YIGSR (from laminin) [134]. Furthermore, polymer functionalized multi-component hydrogel networks of ECM molecules have been designed to better mimic the physical properties or biochemical complexity of native tissues [135]. Tri-component networks composed of collagen, methacrylate-modified chondroitin sulfate, and HA [135]; methacrylated HA within a fibrin hydrogel [136]; and interpenetrating polymer networks of photocrosslinked HA and collagen [137] are all examples of such multi-component hydrogels. The main advantage of multi-component hydrogels over conventional single-component ECM hydrogel is that multi-component ones may better mimic the biochemical and/or mechanical complexity of ECMs in native tissue. However, a limitation is that multi-component hydrogels may be more difficult or time consuming to fabricate consistently, compared to single-component ECM hydrogels.

Decellularized ECM can also be used to produce hydrogels through enzymatic solubilization of the ECM, followed by neutralization to physiological pH and temperature [138,139]. ECM hydrogels derived from decellularized tissues can recapitulate aspects of physiological tissues or stem cell niches. Accordingly, they are an attractive substrate for 3D organoid culture to promote the proliferation and differentiation of stem cells [130,140] as well as tissue morphogenesis [141]. The biochemical, topological, and viscoelastic properties of ECM hydrogels [142] depend on the tissue from which they are derived along with the decellularization procedure. ECM hydrogels have been proposed to have the potential to promote endogenous repair of the myocardium [143] as they mitigate the expression of pro-inflammatory and pro-apoptotic genes and promote blood vessel formation and recruitment and differentiation of stem and progenitor cells in the heart.

**Bioprinting:** In order for hydrogels to reproduce the anatomy and multicellular arrangement of human cardiovascular tissue [144,145], biofabrication strategies have been shown to be useful. 3D bioprinting techniques such as inkjet, microextrusion-based, and laser-assisted bioprinting regulate effective control of biomaterial disposition. With recent advancements, some bioprinting approaches also incorporate topographical and biochemical cues [141]. Various 3D bioprinting techniques have respective strengths and limitations (Table 4). Biomaterial inks used in bioprinting should support cellular viability, proliferation, maturation, and differentiation in the engineered cardiovascular tissue construct [146]. Moreover, the inks should also possess mechanical properties and printability such as viscoelasticity, shear thinning property, and tissue maturation efficiency post bioprinting [147] for structural mimicry for cardiovascular tissue-specific anatomy. Zhang et al. developed a co-axial 3D bioprinting method having the bio-ink as GelMA/alginate blended hydrogel seeded with ECs at the inner needle and calcium chloride at the outer needle for ionic crosslinking, followed by seeding cardiomyocytes onto the engineered scaffold to produce endothelialized myocardium [147]. In two weeks of culture, a layer of endothelium was formed surrounding the microfibers. These examples demonstrate the advancement of increasingly complex geometries using bioprinting strategies.

A strength of 3D bioprinting is that it allows the rapid fabrication of functionalized tissue with physiologically relevant architecture and microenvironmental cues. Additionally, 3D bioprinting is capable of handling a large number of cells while also being scalable. However, it is difficult to replicate native human cardiovascular tissues/organs without a biomaterial or “bio-ink” that enables precise fabrication and promotes cellular behavior. ECM-based biomaterials are promising bio-inks because they facilitate the engineering of functional cardiovascular tissue by supporting biological activity, tissue resilience, and structural mimicry. Decellularized ECM bio-ink contains biochemical cues from the original native ECM microenvironment required for cellular proliferation and growth with the appropriate proportions of ECM proteins. Cell-specific bio-inks could be tailored for bioprinting of specific cell types to stimulate physiological mechanisms in cellular models. The convergence of ECM with 3D printing technology holds great potential for printing complex bio-scaffolds especially biomimetic 3D structures. With these advancements, a highly organized and functional cardiovascular tissue (i.e., myocardium, heart valve, and vasculature) can be engineered that is structurally and functionally more similar to native cardiovascular tissue that is suitable for transplantation, drug development, and disease modeling.

Each of the biofabrication strategies to generate functional tissue constructs depend on bio-inks that encapsulate cells, and their requirements depend on the printing modality. For example, inkjet bioprinting requires relatively lower viscosities to prevent clogging and low conductivity the prevent cellular heat damage. Extrusion-based biofabrication processes can accommodate high viscosities but require shear thinning materials to prevent cellular mechanical damage [148,149]. In extrusion-based fabrication processes, the bio-ink is extruded continuously through a deposition nozzle. Therefore, low viscosity is desirable during extrusion to avoid high shear stress and potential clogging. Upon deposition, a high viscosity rate is needed to maintain shape and high print fidelity to preserve high printing precision such as thermo-responsive gelation of gelatin that retains its shape of printed structure [150]. However, gelatin is not used alone in biofabrication as its reversible sol-gel transition can affect printing temperature and viscosity [151]. Similarly, PEG solution has low viscosity and is too soft to keep its shape post printing [152,153]. Bio-inks containing cells must also remain viable during the fabrication process [154]. Hydrogels such as agarose maintain their structural integrity through high polymer concentration. However, the resulting high viscosity can affect cell viability; hence, agarose hydrogels are used as sacrificial structures [148]. High structural integrity is an indicator of success in applications such as vascular grafts [148,155].

Biomaterials have physical, chemical, and biological properties that influence the bio-fabrication process. These include viscosity, shear thinning, viscoelasticity, gelatin kinetics, biocompatibility, biodegradation, and hydration degree [156]. The rate of gelatin affects the print fidelity by determining the speed of hydrogel crosslinking after printing [148]. Advanced biomaterials such as hydrogels use multiple approaches to improve printability and cytocompatibility. For example, biomaterials such as supramolecular hydrogels, interpenetrating network and nanocomposites are designed with shear thinning characteristics and have lower viscosities at high shear rates of extrusion. Post extrusion, an increase in viscosity results in high print fidelity and cell viability. Biodegradation of hydrogels can occur enzymatically (i.e., collagen, gelatin), hydrolytically (i.e., polyester), or through ion exchange (i.e., alginate) [155]. The degradation kinetics of hydrogels modulate ECM production and remodeling.

ECM-based hydrogels can be modified to improve their strength, shape integrity or resistance to rapid degradation. Hydrogels made from matrix molecules (i.e., a gel formed from myocardia matrix) have been reported to have low stiffnesses of 5–10 Pa at 1 Hz [157]. Low mechanical integrity and modulus can negatively affect cell adhesion, migration, and signal transport. Christman and collaborators showed that the addition of PEG to the either NHS or PEG diacrylate can increase the storage modulus of the gel (5–30 Pa), compared to ECM gel (5 Pa) [157]. The incorporation of PEG allowed for tunable degradation, compared to ECM gel that exhibited degradation times 2–3 times faster than the hybrid gel. Cell studies reveal that cellular adhesion, migration, and encapsulation of myocardial-PEG-NHS or acrylate hydrogels was efficient compared to ECM gels that showed very low cell encapsulation efficiency [157]. Mintz and collaborators reported that the storage modulus of polycaprolactone (PCL) porous scaffolds injected with HA hydrogel [158] was higher than that of hydrogel alone and lower than the PCL scaffold alone. However, the hybrid biomaterial did have a noticeably different Young’s modulus compared to the PCL scaffold, demonstrating that hybrid scaffolds can provide high stiffness characteristics in compression and tension (PCL component), while concurrently exhibiting viscoelastic properties of hydrogels (HA).

**Spatial Patterning:** Each organ and tissue has a distinct ECM composition and ultrastructure that modulates cell behavior [163]. This can be recreated by micropatterning of ECM components onto synthetic materials [164]. Photolithography or light-based patterning can be used for differential ECM protein deposition onto a substrate. It has the potential to achieve a resolution of 500–5000 µm [165], but the high cost of photolithographic equipment and clean room maintenance [166] are a few limitations of the technique. On the other hand, elastomeric stamping techniques are procedurally simple and inexpensive [167], have improved control mechanisms, can incorporate microchannels and microfluidics, and enable patterning of gradients of ECM components. Additionally, the electrospinning technique employs a current to induce the formation of nano- to micro-scale fibers that can be arranged in parallel alignment [168,169]. Furthermore, nanofiber lithography can be used to fabricate fibrous nanopatterned scaffolds with a resolution of 250–1000 nm [170] and regulates cell adhesion through modulation of integrin expression. In another approach, we developed a facile shear-based approach for the fabrication of spatially nanopatterned collagen scaffolds that support the parallel organization of vascular ECs and SMCs [171].

Cellular signaling pathways are regulated by the composition, topography, and mechanical properties of micropatterned ECM substrates. For example, the shape and phenotype of macrophages can be modulated by the elasticity and rigidity of micro-patterned substrates with fibronectin without exogenous cytokines [172]. Substrate elasticity can modulate actin polymerization and activation of stretch sensitive ion channels that can mediate changes in macrophage gene expression and cytokine secretion. Angiogenesis has similarly been shown to be modulated by micropatterning as strong mechanical forces [173,174], as the convex part of micropatterned vessel walls promote preferential formation of blood vessels. We previously demonstrated that parallel-aligned micropatterned channels as well as nanopatterned collagen scaffolds promote the organization and migration of ECs [175]. Even when laminar flow was applied to EC-seeded parallel-aligned nanofibrillar collagen scaffolds orthogonal to the direction of collagen patterning, the cells preferentially remained organized along the direction of spatial patterning. Mechanical forces can modulate very important cellular [175,176,177,178] and biological functions such as differentiation [179], apoptosis [180], gene expression [181], and RNA processing [182], emphasizing the critical role of ECM structure, topography, and mechanics for tissue remodeling. Even though recent advancements in the imaging and data analysis techniques have helped reveal the remarkable ECM architecture, one of the challenges in the exact recreation of the ECM structure by spatial patterning is the detailed mapping of the native ECM topography.

## 6. Translational Applications of ECM-Based Biomaterials

### 6.1. Engineered Vascular Grafts

Autologous blood vessels are the gold standard for bypass graft surgeries. However, their use is limited due to the lack of suitability vessels, especially in diseased patients, owing to low quality and high failure rates [183,184,185,186]. Alternatively, tissue-engineered small-diameter vascular grafts made from synthetic or natural polymers have not demonstrated adequate results, comparable to autologous grafts. Synthetic polymer grafts such as polyethylene terephthalate/Dacron and polytetrafluoroethylene/Teflon (ePTFE) have high failure rates comparable to small-diameter vascular grafts due to the lack of an endothelium, thrombosis, and intimal hyperplasia [187,188,189,190]. In contrast, scaffolds derived from purified ECMs such as collagen and fibrin are promising due to being biocompatible and promoting endothelialization [191,192,193].

Three different approaches have been employed for using ECM in vascular graft applications. These consist of cell-derived ECM, extracted 2D tissue, and cannular tissue. Cell-derived grafts are created from a monolayer or cannular-shaped culture of cells. After cells deposit ample ECM, the cells are removed, leaving behind decellularized ECM. The extracted 2D and cannular tissues produce grafts by decellularization and surface modification approaches. The grafts can be implanted in an acellular fashion or cultured with autologous ECs. For example, Niklason et al. utilized the cell-based ECM approach for small-diameter vascular grafts. The investigators seeded and cultured SMCs (bovine [194,195], porcine [194,196], canine [197], and human [197,198]) and human MSCs onto a polyglycolic acid (PGA) mesh scaffold for 8–10 weeks. During the culture period, the grafts demonstrated appropriate vessel wall thickness, suture retention strength, burst pressure, and collagen content [194,195]. These grafts have shown good long-term patency in vivo in canine and baboon models [197]. Further, these grafts showed promising patency results in clinical trials for hemodialysis access in patients with renal disease—80% patency at 2 years [198]. Despite the positive patency results, the degradation components of PGA caused a phenotypic change of SMCs from contractile to synthetic, along with a lower expression of contractile proteins myosin, SMA, and calponin [101,194,195,199]. The phenotypic shift results in intimal hyperplasia and atherosclerosis [200,201,202,203,204]. Table 5 summarizes the different approaches to develop cell-derived extracellular matrix based vascular grafts.

The most commonly used 2D scaffold for small-diameter vascular grafts is porcine small intestine submucosa (SIS). SIS has superior mechanical properties (burst pressure and compliance) analogous to native vessels, but low patency rates [205,206]. Decellularized SIS cultured with human vascular ECs showed higher proliferation rates and contractile morphology, compared to bare ePTFE and Dacron scaffolds [207]. SIS scaffolds treated with fibrinogen and thrombin showed EC culture and graft patency ex vivo [208]. The grafts were treated with heparin and pre-seeded with SMCs and implanted into sheep for 90 days. After the culture period, the grafts exhibited lumens with no sign of intimal hyperplasia or clotting [209]. The grafts with pre-seeded SMCs had a higher concentration of collagen content, indicating cellular maturation [209]. Table 6 summarizes the 2D tissues for vascular grafts.

Decellularized porcine conduits require surface treatments to enhance hemocompatibility properties of the grafts. Ma and collaborators cultured decellularized porcine aortas with autologous ECs and confirmed long-term patency for 3 months [212]. Li and collaborators studied the in vitro efficacy of decellularized porcine carotid artery conduits cultured with hMSCs derived ECs and SMCs [213]. Immobilization of heparin on decellularized porcine carotid artery conduits exhibited anti-thrombogenic properties [214]. Table 7 summarizes cannular tissue studied for small diameter vascular grafts.

### 6.2. Cardiac Patches

In order to overcome the limitations of traditional cellular therapy including the availability of oxygen and nutrients, the fabrication of thick 3D constructs of defined geometry and complexity and well-aligned cellular networks has been studied extensively [216]. Ventricular function lost in myocardial infarction can be regained by replacing the necrotic tissue with a tissue engineered “cardiac patch” [217,218]. Bioengineered functional cardiac tissue composed of CMs has been studied extensively for myocardial regeneration potential and in vitro tissue remodeling. One of the challenges in designing a functional cardiac tissue is well-defined cell contraction and alignment [219]. Cardiac patches should ideally be electrically conductive, mechanically robust and elastic, and prevascularized for functional integration into organ architecture resulting in improved contraction potential. Zhang and collaborators bioprinted a scaffold using an EC-laden bio-ink with sodium alginate and gelatin/methacylate (GelMA) seeded with CMs to create an endothelialized myocardium [220]. A rigid 3D structure was formed as a result of crosslinking of alginate with calcium ions followed by UV of the GelMA. Endothelial cells migrated towards periphery of the scaffold fibers and formed a confluent layer. Cardiomyocytes seeded into the scaffold with controlled anisotropy formed an aligned myocardium with contractions in a synchronous manner. This developed a functional myocardium with an interlacing endothelium and well-aligned cardiomyocytes.

Extensive research has been performed to replace infarcted cardiac tissue with tissue-engineered cardiac patches made of biocompatible and bioabsorbable materials such as purified ECM molecules and heterogeneous mixtures of ECM components [221]. Jang and collaborators created a 3D prevascularized stem cell patch through spatial organization of cardiac progenitor/MSCs using decellularized ECM bio-ink [222]. The cardiac patch was shown to decrease cardiac remodeling and fibrosis and promoted cardiomyogenesis and neovascularization at the injured myocardium post transplantation. Gao et al. 3D-printed an EPC/atorvastatin-loaded PLGA microspheres laden bioblood vessel and bio-ink composed of vascular tissue-derived ECM and alginate [223]. The engineered tissue revealed enhanced viability, proliferation, and differentiation of endothelial progenitor cells (EPCs) and endothelialization in vitro. In a nude mice hind limb ischemia model, the bioblood vessel (BBV)-based method showed significantly improved EPC function and recovery rates of ischemic injury. These studies illustrate the advancement of cardiac patches for preclinical testing.

### 6.3. Organ-on-a-Chip

Organ-on-a-chip engineering involves the microfabrication of artificial organs with tissue-engineered ECM and various types of cells to recapitulate morphogenesis, differentiation, and functions of the organ. Organ-on-a-chip models have the potential replicate key aspects of human physiology and revolutionize disease modeling, pharmacological studies, and pre-clinical drug development. The success of these tissue models critically depends on the functional assembly and human cell maturation that are building blocks on organ-on-a-chip systems, for example, assembly of human stem cell-derived CMs in a controlled microenvironment into a functional cardiac tissue.

Integration of 3D printing into organ-on-a-chip engineering can facilitate organ microfabrication with heterogeneity, a desired 3D cellular arrangement, tissue-specific functions, desired cellular arrangement, and mechanical and electrical components. ECM components have been extensively studied in vitro and in vivo for organ-on-chip engineering. The most abundant ECM component, the collagen fibrillar structure, assembles into a viscoelastic gel under physiological pH, temperature, and ionic strength conditions that provides a cell-adhesive, supportive, and structural network. The mechanical properties of the gel can be tuned by adding crosslinkers (e.g., 1-ethyl-3-(3-dimethylaminopropyl)carbodiimide and n-hydroxysuccinimide) that makes collagen an attractive bio-ink for organ-on-a-chip applications [224]. Fibrin has been used in maintaining the 3D shape of the printed constructs due to its rapid gelation properties. Hinton and collaborators successfully printed whole brain and heart structures by dispensing collagen hydrogel containing fibrin and cellular components into a gelatin slurry bath with thrombin [225]. In another example, Olya and collaborators developed a 3D human cardiac fibrosis-on-a-chip (hCF-on-a-chip) platform that recapitulated the distinctive characteristics of cardiac fibrosis and provided proof-of-principle for phenotypic analysis of a drug for treatment of idiopathic pulmonary fibrosis [226]. The group designed a human hCF-on-a-chip model to screen for possible biomarkers of human cardiac fibrosis. Additionally, the group used human iPSC-derived cardiomyocytes to study the pathophysiologically relevant capabilities of the human hCF-on-a-chip device, including cardiomyocyte functional analysis to unravel the understanding of disease progression for treating heart failure and cardiac fibrosis. dECM has been widely used in reproducing the natural environment of cells in native tissues [227]. These studies illustrate the applications of ECM-based biomaterials for organ-on-a-chip applications.

## 7. Future Perspectives and Conclusions

Despite the benefits of biocompatibility and low immunogenicity of ECM-based biomaterials for cardiovascular tissue engineering and regenerative medicine, a number of limitations hinder their clinical translation. First is the limited ability to control their mechanical properties. As an example, the structure of collagen scaffolds, either crosslinked or decellularized, is relatively fragile and temperature sensitive. Therefore, these biomaterials are generally not able to withstand physiological burst pressures. Improving mechanical properties by chemical modification (i.e., methacrylation [228,229]) or incorporation of polymers [230] and other proteins [231] can be beneficial. Second, the properties and composition of decellularized ECM may be inconsistent due to batch-to-batch variability [232]. To reduce batch variations, a single batch using pooled sources can reduce some of the variability. Another limitation is the inability to sterilize the scaffolds using conventional high-heat methods. Alternative sterilization methods using low-dose gamma irradiation (γ-ray) alters the molecular structure and decreases the mechanical and enzymatic resistance of the collagen scaffold [233,234,235,236]. Additionally, ECM-based biomaterials have limited tunability of their degradation kinetics. For example, purified non-crosslinked collagen hydrogel injected into the myocardium persisted only for several days [237], whereas crosslinked high-density collagen scaffolds only partially degraded after seven weeks in porcine muscle [238].

The design of multi-component ECM scaffolds depends on the size and nature of the components. At the molecular level, integrating proteins and polysaccharides into a polymer synthetic hydrogel can impart biological functions and tunability in molecular architecture, chemical composition, and mechanical properties. At the microscopic level, the addition of micro or nano particles into hydrogels provides biological functions and mechanical characteristics. It allows for the spatiotemporal release of biologically active compounds for desired cellular functions. While hybrid approaches offer unprecedented opportunities for mimicking the complex native environment, there are challenges that need to be addressed These materials are inherently heterogenous, with chemical and structural variations that can complicate the engineering of hybrid scaffold. As the desired biological complexity increases, stability and consistency become challenging to achieve. New approaches for maintaining protein structure and function and particle synthesis will help design well-integrated hybrid materials with structural stability. Finally, immunogenic properties of new materials need to be studied concomitant with design of tissue engineered constructs. The translation of these advances to address biological challenges offers promising applications of these hybrid materials in tissue engineering.

Despite ECM-based biomaterials generally having low immunogenicity, it has been reported that the residual nucleic acids and cell membrane epitopes (i.e., galactose-alpha-1, 3 galactose) can trigger adverse immune response post implantation [10,11]. Modulating the immune response by incorporating immunosuppressive molecules (i.e., TGFβ1, IL-10 [239]) anti-Fas antibodies [240,241], or gene-editing strategies [242] may partially address the concerns of immunogenicity. Despite these limitations, ECM-based biomaterials remain promising, and we anticipate the rise of ECM-based biomaterials being used in clinical applications of cardiovascular regeneration.

## Figures and Tables

**Table 1:** Effect of ECM-based biomaterials on cardiovascular lineages.

ECM	ECM-Based Biomaterials	Model	Cardiovascular Tissue Engineering Advantages	Ref.
Collagen	COL I	Murine	Cardiomyocyte differentiation, maturation and contractile function	[90]
Collagen	ESC and iPSC/COL IV	In vitro	Differentiation of induced pluripotent stem cells (iPSs) into cardiomyocytes of contractile function.	[91]
Collagen and Fibrin	COL 1/Fibrin	In vitro	Improved physical property, cardiac tissue compaction	[63]
Collagen and Elastin	COL 1/Elastin	In vitro	Enhanced elasticity, maturation of valve interstitial cells and valve ECs.	[65]
Fibrin	Fibrin	In vitro	Cardiomyocyte proliferation and cardiac regeneration	[92]
Collagen	COL 1/Growth factors/Matrigel^TM^	In vitro	Cardiomyocyte differentiation and maturation	[93]
HA	HA	In vitro	Attenuates cardiac fibrosis and promote cardiac muscle tissue regeneration	[94,95]

Abbreviations: ESC (embryonic stem cell); iPSC (induced pluripotent stem cell); COL 1 (collagen I); COL4 (collagen IV); EC (endothelial cell); HA (hyaluronic acid).

**Table 2:** Decellularized ECM-based biomaterials and cardiovascular response.

Tissue/Organ	Decellularization Method	Model	Vascular Response	Ref.
Adult Porcine Heart	Pulsatile retrograde aortic perfusion	Porcine	Chicken cardiomyocytes were cultured. ECM retained collagen, elastin, glycosaminoglycans, and mechanical integrity.	[96]
Porcine Whole Heart	Perfusion of Trypsin/EDTA and Triton-X100/deoxycholic acid (DCA)	Porcine	ECM retained elastin, collagen, and proteoglycan	[97]
Rat Heart Adult	Three decellularization solutions were used: (1) SDS/TritonX100- v/s (2) Trypsin plus Triton/DCA v/s (3) SDS/DCA/saponin	In vitro	C2C12 myoblasts were seeded in vitro. ECM retained laminin in all groups, elastin in groups 1 and 2, collagen in groups 1 and 3.	[98]
Porcine Aortic Valves	Triton X-100	In vitro	ECs were seeded. EC mediated ECM deposition was observed.	[99]
Human Umbilical Artery	SDS treatment	In vitro/In vivo	In vitro EC cell seeding and implantation in rats. EC culture growth was sustained for 8 weeks, ECM preserved.	[100]
Tissue Engineered Porcine Vessels	SDS	In vitro/Porcine	Pre-seeding of EC progenitor cells and implantation in porcine carotid artery. No clotting observed. No signs of intimal hyperplasia.	[101]
Bioartificial Tissue Engineered Heart	Coronary perfusion-based whole organ decellularization	In vitro/rat model	Recellularization with neonatal cardiac cells and rat aortic ECs. Constructs exhibited contractile function and EC culture growth.	[77,78]
Neonatal Mouse Heart	Liquid nitrogen, erythrocyte lysis, and DNA/RNA removal	In vivo model of acute MI	nmECM showed improved heart function compared to adult heart derived ECM, cardiac repair after MI	[79]
Cardiac Porcine Hearts	SDS	In vitro	cdECM promotes human cardiac fibroblast culture and human iPSC-derived cardiomyocytes.	[102]
Porcine Cardiac Tissue	SDS	In vivo	Decellularized porcine myocardial extracellular matrix (dECM)-reduced graphene oxide hydrogel promoted increased expression of genes that regulated contractile function.	[103]
Porcine Heart	SDS	In vitro	Enhanced maturation of cardiomyocytes (isolated from neonatal rats) in hdECM	[104]
Rat Heart Tissue	Ionic and non-ionic detergents	In vivo	(AdMSCs) promoted increased cardiomyocyte-specific gene expression for 2–4 weeks.	[105]

Abbreviations: SDS (sodium dodecyl sulfate); EC (endothelial cells); ECM (extracellular matrix); DCA (deoxycholic acid); MI (myocardial Infarction); nmECM (ECM derived from neonatal mouse hearts); cdECM (cardiac decellularized ECM); iPSC (induced pluripotent stem cell); dECM (decellularized ECM); hd ECM (heart tissue-derived ECM); (AdMSCs) adipose tissue-derived mesenchymal stem cells.

**Table 3:** ECM-mediated endothelial differentiation and function.

ECM Component	Model	Endothelial Cell Response	Ref.
Laminin	In vitro	Differentiation of embryonic stem cells into functional endothelial progenitor cells.	[114]
Laminin	In vitro	Laminin facilitates in vitro 3D vascular network formation by promoting uptake of VEGF by ECs.	[115]
COL IV- multi-component ECM	In vitro	Improved endothelial differentiation of human induced pluripotent stem cells.	[118]
Fibronectin	In vitro	EC growth and proliferation was supported by fibronectin coating on vascular grafts.	[121]
RGD-modified HA	In vivo	EC migration and proliferation, formation of functional vasculature	[122]
dECM	In vitro	endothelial progenitor cells encapsulated in blood vessel-derived dECM exhibited higher proliferation capability and enhanced vascular network formation.	[123]
dECM	In vitro	In vitro EC cell seeding and implantation in rats. EC culture growth was sustained for 8 weeks, ECM preserved.	[100]
dECM	In vivo	Hybrid ECM promoted proliferation and migration of HUVECs, significantly inhibited immune response and calcification, exhibited stability and biocompatibility compared to non-hybrid leaflet.	[124]
Heparin	In vitro	Surfaces covalently immobilized with heparin promoted endothelial cell growth and inhibited SMCs.	[125]
Fb/COL/LA/FN	In vitro	High EC cell densities were achieved in 7 days of culture	[126]
Fibrin Fragment E (FbnE)	In vitro	Increased adhesion and endothelial differentiation.	[127]
COL Coated PCL Membrane	In vitro	Continuous EC monolayer was observed on collagen coated membrane. ECs exhibited filopodia protruding from lamellipodia in the junctional areas on the collagen-coated membranes.	[128]
Matrigel Matrix Comprising of LA, COL IV, Heparen Sulfate Proteoglycans	Ischemic mouse model	Improved neovasculature formation, promote cell growth, proliferation and differentiation of ECs.	[129]
Cardiogel Composed of LA, FN and Interstitial COL I and IV	In vivo	ECM components promote growth of ECs and CMs, spontaneous contractile activity and phenotypic morphological differentiation.	[130]

Abbreviations: VEGF (vascular endothelial growth factor); EC (endothelial cells); ECM (extracellular matrix); DCA (deoxycholic acid); MI (myocardial Infarction); nmECM (ECM derived from neonatal mouse hearts); cdECM (cardiac decellularized ECM); iPSC (induced pluripotent stem cell); dECM (decellularized ECM); hd ECM (heart tissue-derived ECM); (AdMSCs) adipose tissue-derived mesenchymal stem cells; HA (hyaluronic acid); Fb (fibrin); LA (laminin); FN (fibronectin); COL (collagen); PCL (polycaprolactone).

**Table 4:** Advantages and limitations of bioprinting techniques.

3D Bioprinting Technique	Advantages	Limitations	Ref.
Inkjet Bioprinting	Uses thermal, electromagnetic or piezoelectric technology to deposit droplets of “ink” (materials) Rapid printing speeds and high resolution. Capable of printing low-viscosity biomaterials. Availability and ease of replacement of bio-inks. High-cell viability and relatively low cost	Low material viscosity (<10 Pa·s) and low droplet directionality. Lack of precision with respect to droplet size. Requirement for low viscosity bio-ink. Nozzle clogging and cellular distortion due to high-cell density. Low mechanical strength. Inability to provide continuous stream of material.	[159]
Micro-Extrusion	Ability to print biomaterials with high cell densities (higher than 1 × 10^6^ cells mL^−1^) comparable to physiological cell densities. Can produce continuous stream of material. Can successfully print high viscosity bio-inks such as polymers, clay-based substrates.	Low printing resolution (>100 µm) and slow printing speeds. Loss of cellular viability and distortion of cellular structure due to the pressure to expel the bio-ink.	[160]
Laser-Assisted Bioprinting: SLA and LIFT	Rapid printing speeds and ability to print biomaterials with wide range of viscosities (1–300 mPa/s). High degree of precision and resolution (1 cell/droplet). Can successfully print high density of cells 10^8^ mL^−1^	Time consuming: need to prepare reservoirs/ribbons. Lower cellular viability compared to other methods. Loss of cells due to thermal damage. SLA requires intense UV radiation for crosslinking process. Requires large amount of material. High cost. Long post processing time and fewer materials compatible with SLA.	[161,162]

Abbreviations: LIFT (laser-induced forward transfer); SLA (stereolithography SLA).

**Table 5:** Cell-derived ECM strategies for engineering vascular grafts.

Material	Treatment	Model	Vascular Graft Response	Ref.
PGA Scaffold	SMC	Bovine	Grafts exhibited goof vessel wall thickness, burst pressure, and collagen content.	[194,195]
PGA Scaffold	SMC	Canine	Grafts exhibited good long-term patency for 8–10 weeks.	[197]
PGA with Fibrinogen and Thrombin	SMC s and ECs derived from hiPSC	Porcine	Grafts exhibited endothelial differentiation.	[194,196]
PGA Scaffold	MSCs	In vitro	Grafts exhibited superior mechanical properties and cellular growth.	[198]
Fibrin Gel	Human fibroblast (hDFs)	Baboon	Grafts exhibited higher patency rates of >80%.	[196]

Abbreviations: SMC (smooth muscle cells); EC (endothelial cells); hiPSC (human induced pluripotent stem cells); hDF (human dermal fibroblast); MSC (mesenchymal stem cells); PGA (polyglycolic acid).

**Table 6:** Two-dimensional scaffolds for vascular grafts.

Material	Treatment	Model	Vascular Graft Response	Ref.
SIS	EC culture	In vitro	Grafts exhibited higher EC proliferation and cobblestone morphology.	[207]
SIS	Pre-seeded with SMC and fibrinogen/thrombin	Porcine	Grafts exhibited endothelial cell attachment and graft patency.	[208]
SIS	Heparin	Sheep	Grafts exhibited lumens with no sign of clotting or intimal hyperplasia.	[194,196]
Pericardium	MSCs	Bovine	ECM and growth factors facilitated differentiation into ECs.	[210]
Porcine SIS Tubes	Heparin/VEGF	Sheep	Grafts exhibited long term patency rates for 3 months with a confluent endothelium and no signs of thrombosis.	[211]

Abbreviations: SMC (smooth muscle cells); EC (endothelial cells); SIS (small intestine submucosa); MSC (mesenchymal stem cells); VEGF (vascular endothelial growth factor).

**Table 7:** Cannular tissues for vascular grafts.

Material	Treatment	Model	Vascular Graft Response	Ref.
Carotid Artery	Autologous EC	Porcine	Grafts exhibited patency rates of >90% for 6 months.	[212]
Carotid Artery	MSCs	Porcine	ECM and growth factors cause differentiation of MSCs into ECs.	[213]
Carotid Artery	Heparin	Porcine	Grafts exhibited lumens with no sign of clotting/thrombus.	[214]
Carotid Artery	MSCs	Porcine	ECM and growth factors cause differentiation of MSCs into SMCs.	[213]
Porcine Aorta	Autologous ECs	Canine	Grafts exhibited long term patency rates for 3 months.	[210]
Aorta	Heparin/VEGF	Canine	Grafts exhibited patency of >90% post 2 years.	[214]
Porcine Pericardium Scaffold	Fibrin mesh/Heparin/VEGF	Porcine	Grafts exhibited potential to accelerate in situ endothelialization.	[215]

Abbreviations: SMCs (smooth muscle cells); ECs (endothelial cells); MSCs (mesenchymal stem cells); VEGF (vascular endothelial growth factor).
